# A Case of Postoperative Lymphatic Ascites in Endometrial Cancer Spontaneously Resolving After Approximately One Year of Watchful Waiting

**DOI:** 10.7759/cureus.78546

**Published:** 2025-02-05

**Authors:** Keisuke Takefushi, Yuji Tanaka, Tsukuru Amano, Shunichiro Tsuji, Takashi Murakami

**Affiliations:** 1 Department of Obstetrics and Gynecology, Shiga University of Medical Science, Otsu, JPN

**Keywords:** endometrial cancer, gynecology, lymphangiography, lymphatic ascites, lymphorrhea, pelvic lymphadenectomy, pleuroperitoneal communication, postoperative complication, postoperative lymphatic leakage, watchful waiting

## Abstract

Postoperative lymphatic ascites following lymph node dissection for gynecologic malignancies is not uncommon, although in most cases it resolves spontaneously within two to three weeks, or at most within four months. We present the case of a 73-year-old woman who underwent total hysterectomy with bilateral salpingo-oophorectomy and pelvic and para-aortic lymph node dissection for endometrial cancer. She subsequently developed a large volume of ascites, which was diagnosed as lymphatic ascites based on biochemical analysis of the ascitic fluid, cytological examination, and CT findings. An initial watchful waiting strategy was ineffective, and at eight months postoperatively, lymphangiography with Lipiodol was performed both for diagnostic and therapeutic purposes. Although a lymphatic fistula was identified, no therapeutic benefit was achieved, and conservative management was continued. Because the patient exhibited no signs of infection, maintained adequate oral intake, did not require frequent paracentesis or cell-free and concentrated ascites reinfusion therapy (CART), and was able to receive scheduled adjuvant therapy without delay, invasive surgical intervention was avoided. Ultimately, the ascites resolved spontaneously approximately one year after surgery. Mild lower extremity lymphedema was noted thereafter, but no tumor recurrence was observed. This case demonstrates that even if lymphatic ascites persists for as long as 11 months postoperatively, it may still resolve under extended watchful waiting.

## Introduction

Various terminologies have historically been used to describe postoperative lymphatic leakage following pelvic lymph node dissection. In a large-scale review, postoperative lymphatic leakage was categorized based on the nature of the drainage fluid, with “lymphorrhea” and “chylorrhea” proposed as distinct entities. Lymphorrhea is further subdivided into localized collections (lymphocele) and non-localized accumulations (lymphatic ascites) [1]; this classification is now widely adopted.

To diagnose postoperative lymphatic leakage, biochemical and cytological analyses of the aspirated fluid are essential to exclude malignant ascites, intra-abdominal hemorrhage, urinary ascites secondary to bladder injury, and infectious exudates [1]. Although no strict diagnostic criteria distinguish lymphorrhea from chylorrhea, these conditions are often differentiated by fluid color (yellow and transparent vs. milky white) and triglyceride levels (low vs. high).

The reported incidence of lymphatic ascites after lymph node dissection for gynecologic malignancies varies from 1.8% [2] to 2.5% [3] and 4% [4]. Regarding preventive measures, some studies have shown that selecting specific surgical devices may reduce the incidence of postoperative lymphatic ascites [5], while others have demonstrated no difference [6]. When lymphatic fluid leaks into a confined space formed by postsurgical adhesions, it becomes a lymphocele; compression of adjacent organs (e.g., urinary tract or bowel) or infection may ensue. In contrast, most cases of lymphatic ascites are asymptomatic and resolve spontaneously within two to three weeks [1]. In a study of 300 patients who underwent lymph node dissection, 12 developed lymphatic ascites, and the median time to resolution was 44 days (range: 9-99 days) [4]. A retrospective analysis of 3,427 lymph node dissections identified 63 cases of lymphatic ascites, which resolved with conservative treatment, including drainage and nutritional support, in a median of 10 days (range: 3-56 days) [2]. Even with broadening the scope to include chylous ascites, the median resolution time in most reports does not exceed 11 days [7], and the longest reported case resolved in about five months [8].

Herein, we report a case of lymphatic ascites that spontaneously resolved after approximately one year of postoperative watchful waiting.

## Case presentation

A 73-year-old multiparous postmenopausal woman was referred to our hospital with a diagnosis of endometrial cancer. On her initial visit examination, her weight was 44 kg (body mass index, 20), height was 148 cm, and her medical history included diabetes mellitus. CT and magnetic resonance imaging (MRI) revealed deep myometrial invasion (≥50% of the myometrium) and enlarged lymph nodes (>10 mm in diameter) in the region of the common iliac artery bifurcation. Histological findings of the endometrial biopsy revealed grade 1 endometrioid carcinoma. Based on these findings, she was clinically categorized as cStage IIIC1 according to the International Federation of Gynecology and Obstetrics (FIGO) 2008 criteria. She underwent an open total hysterectomy with bilateral salpingo-oophorectomy, pelvic lymph node dissection, and para-aortic lymph node dissection. A total of 44 lymph nodes (29 from the pelvis and 15 from the para-aortic region) were removed, of which seven were metastatic. The caudal ends of the suprainguinal nodes were sealed using a vessel-sealing system and electrocautery. The caudal ends of the obturator nodes were ligated with absorbable sutures and cauterized with a vessel-sealing device. A closed-suction (pleated) drain was placed in the pelvic cavity. The final pathology confirmed FIGO 2008 Stage IIIC2 endometrial cancer (endometrioid carcinoma, grade 1) with negative peritoneal cytology, pT2N2M0, positive lymphovascular invasion, and no venous invasion. Daily drainage volume was 187 mL on the day of surgery, 360 mL on postoperative day 1, 460 mL on day 2, 1,032 mL on day 3, 820 mL on day 4, and 300 mL on day 5, after which the drain was removed. The patient was discharged on postoperative day (POD) 10.

Following two weeks postoperatively, she developed ascites and weight gain (up to 54 kg). Although her weight temporarily decreased around one month postoperatively, massive ascites reaccumulated at 1.5 months after surgery. The weight gain and ascites accumulation were clinically diagnosed as postoperative lymphatic leakage. Despite the presence of abdominal distension, the patient's performance status remained between 0 and 1, and her laboratory values were acceptable. Considering the risk of postoperative recurrence, adjuvant chemotherapy with doxorubicin and cisplatin was initiated on POD 23, with six cycles administered at three‐week intervals without delay.

**Figure 1:**
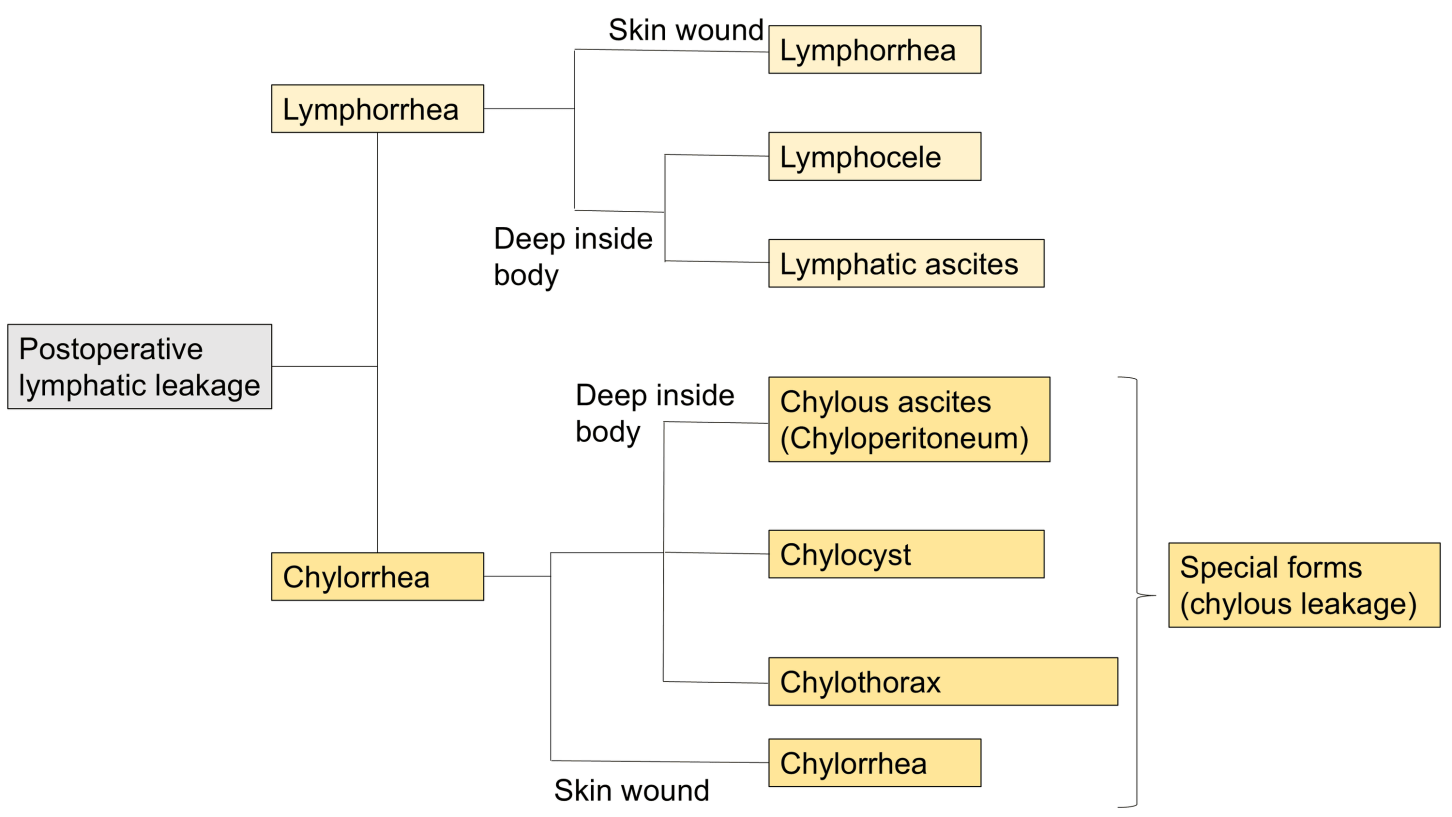
Classification of postoperative lymphatic leakage Modified from [1], which has been published under a Creative Commons Attribution 4.0 License.

Even though postoperative lymphatic leakage is generally expected to resolve with conservative management within four months, the persistent ascites observed at four months, postoperatively, prompted us to consider a broad differential diagnosis, including postoperative lymphatic leakage, malignant ascites, urinary ascites from delayed ureteral or bladder injury, ascites due to doxorubicin-induced heart failure, and ascites resulting from cisplatin-induced renal failure, and to thoroughly evaluate these possibilities. Taking that into consideration, 10 mL of yellow, transparent fluid was aspirated transvaginally from the vaginal stump. Cytology was negative, revealing only abundant lymphocytes, and the ascitic fluid creatinine level was 0.5 mg/dL, suggesting a lymphatic origin and indicating that the ascites was not due to delayed urinary tract injury. Cardiac function on echocardiography was unremarkable, and a serum creatinine level of 0.47 mg/dL effectively ruled out heart failure related to doxorubicin cardiotoxicity as well as renal failure due to cisplatin. Following the diagnostic flow outlined in a previous review, the fluid was classified as “lymphatic ascites” (Figure 1) [1].

**Figure 2:**
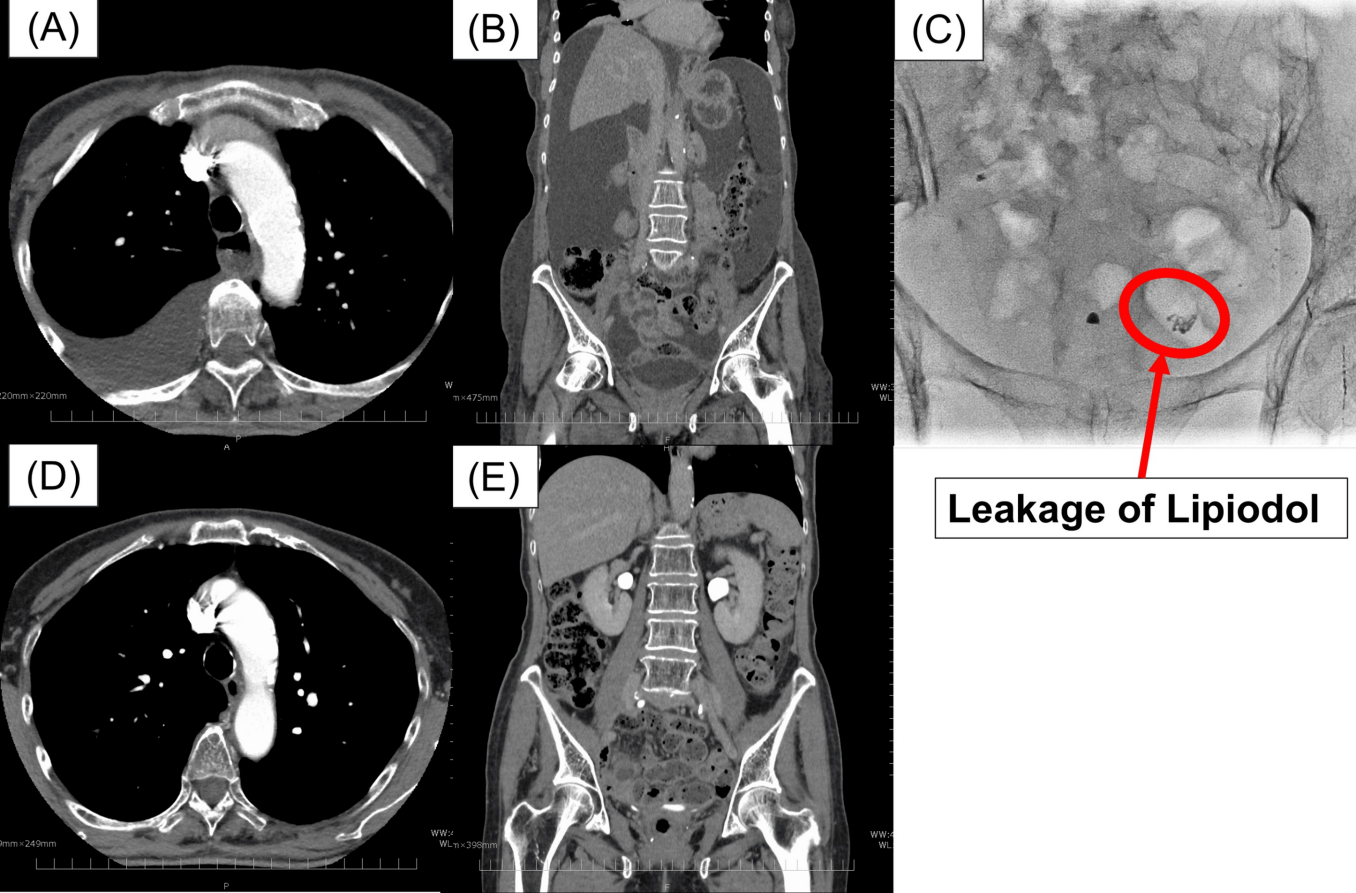
Imaging findings in this case (A) Chest CT at seven months postoperatively shows a right-sided pleural effusion without pleural nodules suggestive of malignant dissemination. (B) Abdominal CT at seven months postoperatively shows massive ascites but no peritoneal nodules suggestive of metastasis. (C) Lymphangiography demonstrates leakage of Lipiodol (red circle) into the abdominal cavity. (D) Chest CT at 18 months postoperatively shows resolution of the pleural effusion. (E) Abdominal CT at 18 months postoperatively shows no ascites.

At seven months postoperatively, although she reported considerable abdominal distension, she showed no signs of infection, remained active with a performance status of 0 to 1, and tolerated oral intake. Follow-up CT showed a large volume of ascites and pleural effusion with an attenuation value of 8 HU, but no evidence of lymphadenopathy, peritoneal nodules, pleural nodules, or visceral metastases (Figure 2). The patient also developed deep vein thrombosis, which may have been partly related to intravascular dehydration secondary to the ascites, and direct oral anticoagulants were initiated. Because she did not require repeated paracentesis or cell-free and concentrated ascites reinfusion therapy (CART), we continued conservative management. From four to eight months postoperatively, her body weight fluctuated between 53 and 57 kg, no lower extremity lymphedema was noted during this period. She was followed up with outpatient visits one to two times per month.

At eight months postoperatively, apart from the initial diagnostic aspiration, no additional paracentesis was performed. Given the prolonged clinical course, we admitted the patient for further evaluation. At that time, her weight was 53 kg. She underwent CART, during which 3,556 mL of ascitic fluid was collected. Of this, 100 mL was submitted for cytological and cell block pathological examination, which revealed predominantly lymphocytes without malignant cells. Ascitic fluid triglyceride and total cholesterol levels were 25 and 73 mg/dL, respectively (Table 1). For both diagnostic and therapeutic purposes, lymphangiography with Lipiodol was performed. Under ultrasound guidance, Lipiodol was injected into the bilateral inguinal lymph nodes, which revealed extravasation of the contrast medium into the abdominal cavity (Figure 2). However, this procedure conferred no reduction in the ascitic fluid volume, and her weight remained around 53 kg thereafter.

**Table 1:** Biochemical and hematological data from blood and ascitic fluid WBC: white blood count; Cre: creatinine; T-chol: total cholesterol; TG: triglycerides; N/A: not applicable

Parameter	Reference range	Preoperative	Lymphatic ascites (7 months postoperatively)	Post-resolution (11 months postoperatively)
WBC (×10^3^/μL)	3.3-8.6	8.1	4.7	7.2
Hemoglobin (g/dL)	11.6-14.8	12.6	8.6	9.4
Serum total protein (g/dL)	6.6-8.1	7.5	7.2	7.8
Serum albumin (g/dL)	4.1-5.1	4.4	3.0	3.4
Serum Cre (mg/dL)	0.46-0.79	0.53	0.63	0.65
Serum CA-125 (U/mL)	0-34	272	169	64
Ascites Cre (mg/dL)	N/A	N/A	0.66	N/A
Ascites T-chol (mg/dL)	N/A	N/A	73	N/A
Ascites TG (mg/dL)	N/A	N/A	25	N/A

**Figure 3:**
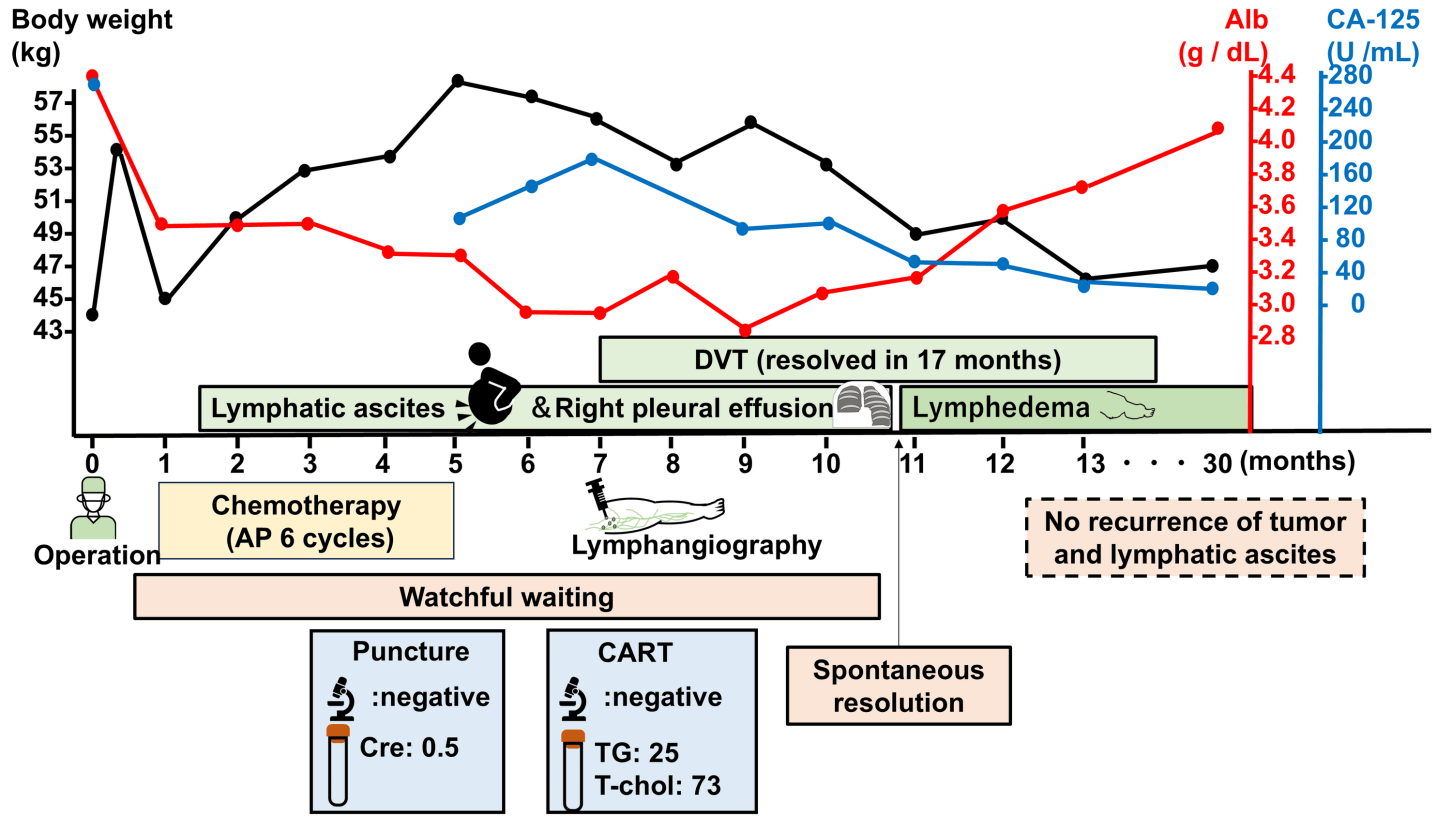
Clinical course of this case Body weight correlates positively with the presence of lymphatic ascites, whereas serum albumin (Alb) inversely correlates with ascites volume. CA-125, which was elevated preoperatively, transiently increased in parallel with ascites and weight gain but normalized once the ascites resolved. DVT: deep vein thrombosis; AP: doxorubicin and cisplatin (every three weeks); CART: cell-free and concentrated ascites reinfusion therapy; TG: triglycerides; T-chol: total cholesterol; Cre: creatinine

At 11 months postoperatively, her weight suddenly dropped to 49 kg, and ultrasonography and CT revealed complete disappearance of the ascites (Figure 2). Concurrently, bilateral lower extremity lymphedema developed. After 2.5 years of follow-up, there has been no recurrence of either the lymphatic ascites or the tumor. The clinical course is summarized in Figure 3.

## Discussion

Here, we reported a case of postoperative lymphatic ascites that spontaneously resolved after nearly one year of postoperative watchful waiting. The initial management of lymphatic ascites typically involves conservative observation to allow spontaneous closure of the lymphatic leak. Although the precise mechanism whereby such ascites resolves remains unclear, persistent elevation of intra-abdominal pressure due to large-volume lymphatic fluid might promote natural sealing of the leaking lymphatic channels. In our case, the eventual disappearance of ascitic fluid coincided with the emergence of lower extremity lymphedema, suggesting that closure of the leaking lymphatic channels redirected lymphatic flow to alternative pathways.

Most reports suggest that lymphatic ascites resolves spontaneously within two to three weeks or by four months at the latest [1,2,4]. In cases with prolonged or refractory leaks, lymphangiography with Lipiodol can be therapeutic [9], as the resultant inflammatory reaction and subsequent fibrosis may help occlude the leak. Surgical interventions include lymphaticovenular anastomosis (LVA) [10] or laparotomic/laparoscopic ligation of leaking lymphatics [11], although these procedures, particularly the latter, are associated with known complications. For chylorrhea, therapeutic measures may include octreotide administration [12], dietary modifications (low-fat diet with medium-chain triglycerides), fasting, and total parenteral nutrition [13,14]. These modalities, however, are generally ineffective for lymphorrhea [1]. Although some have suggested diuretics might be beneficial, evidence remains weak, and concerns about exacerbating thrombotic risk, particularly in cases complicated by venous thrombosis, limit their utility. Thus, in lymphatic ascites, a reasonable management algorithm is to begin with watchful waiting and progress to Lipiodol lymphangiography if conservative measures fail. Even when multiple lymphangiographies prove ineffective, spontaneous resolution can still occur, as reported in a chylous ascites case where ascites resolved after five months of watchful waiting despite repeated imaging procedures [8].

Our experience underscores the notion that even when lymphatic ascites fails to resolve within four months, spontaneous resolution remains possible with continued conservative management, provided that (1) the patient’s activities of daily living are preserved and oral intake is maintained (i.e., no need for repeated paracentesis that could precipitate malnutrition), (2) meticulous attention is given to preventing or managing thrombotic complications, and (3) the patient’s performance status remains stable, thereby enabling adjuvant therapy (chemotherapy or, in some cases, radiotherapy) to be administered without delay. In our patient, no special nutritional interventions were employed because the ascites was classified as lymphorrhea rather than chylorrhea, and she maintained acceptable oral intake throughout her course. Although some reports advocate medium-chain triglycerides or herbal medications [1,15], our patient improved without them.

Interestingly, in this case, ascites volume and body weight varied in direct proportion, whereas increases in serum albumin and hemoglobin inversely correlated with ascites accumulation. Although it is possible that improving nutritional status facilitated resolution of the lymphatic ascites, the patient’s oral intake remained stable before and after chemotherapy, suggesting that once the ascites began to resolve, her nutritional markers improved as a result.

Our patient’s serum CA-125 level, which was elevated preoperatively, decreased once the ascites resolved. Because CA-125 can be produced by the peritoneum, its transient elevation likely reflected peritoneal inflammation caused by the persistent lymphatic ascites rather than tumor recurrence.

We also noted a right-sided pleural effusion in the absence of pleural nodules. This finding suggests the presence of a pleuroperitoneal communication, through which fluid in the abdominal cavity migrates into the pleural cavity under negative intrathoracic pressure. Retrospective studies including patients on peritoneal dialysis estimate the incidence of such communication at 1.6%-1.9%, with right-sided pleural effusion seen in 88% of cases [16,17]. A similar case of isolated right-sided pleural effusion following pelvic lymph node dissection for uterine cancer has also been reported [18].

A key limitation of our investigation is the paucity of published cases describing long-term watchful waiting leading to spontaneous resolution, precluding the formation of a case series. While it is possible that such cases have existed but remained unpublished, our findings nonetheless provide an important clinical insight: even with a protracted time course, lymphatic ascites may resolve without resorting to invasive surgical interventions.

## Conclusions

Here, we reported a case of lymphatic ascites that spontaneously resolved approximately one year postoperatively under watchful waiting. This underscores the value of a patient-centered approach in which clinicians maintain watchful waiting for an extended period when the patient’s condition is stable and no urgent complications arise.
